# Effects of water-rock interaction on the dynamic mechanical properties and energy dissipation of pre-damaged granite

**DOI:** 10.1371/journal.pone.0331541

**Published:** 2025-12-05

**Authors:** Zilong Wen, Huaibao Chu, Luyang Chen, Xiaolin Yang, Haixia Wei, Huazhe Jiao, Jinjin Yang, Fengbin Chen

**Affiliations:** 1 School of Civil Engineering, Henan Polytechnic University, Jiaozuo, Henan, China; 2 Henan Provincial Key Laboratory of Intelligent Construction and Safe Operation of Underground Engineering, Jiaozuo, Henan, China; 3 School of Engineering and Technology, China University of Geosciences Beijing, Beijing, China; Jazan University College of Engineering, SAUDI ARABIA

## Abstract

In deep rock mining, the surrounding rock is often simultaneously subjected to both an initial damage level induced by engineering disturbances and the effects of groundwater, and its stability is crucial for project safety. In this study, we take granite from a mine in Eastern China as the research object and conduct the Hopkinson test, electron microscope scanning test, X-ray diffraction (XRD) test, and elastic longitudinal wave velocity test under varying initial damage level and moisture content conditions. The main purpose of this study is to reveal the variation patterns of the dynamic mechanical properties and energy dissipation of granite specimens under the coupling effect of initial damage level and moisture content. The results show that the dynamic peak stress and peak modulus of granite specimens gradually decrease with increasing initial damage level and moisture content, reaching reductions of 41.6% and 60.6%, respectively, under an initial damage level of 28% and forced saturation. The water-damage weakening coefficient of the specimen increases as the initial damage level rises, making the water weakening effect more pronounced. At a constant initial damage level, the energy dissipation density of the specimen first increases and then decreases as the moisture content increases. As the initial damage level and moisture content increase, the fractal dimension of the specimen increases from 1.801 to 1.865, and the microscopic failure mode transitions from transgranular fracture to mixed-mode fracture, ultimately becoming dominated by intergranular fracture.The research results provide a reference for the stability evaluation and disaster prevention of deep rock mass engineering in water-rich conditions.

## 1 Introduction

With societal development and progress, the exploitation of shallow mineral resources is gradually approaching exhaustion, and the exploitation of deep mineral resources is steadily increasing [1]. During deep mining, engineering disturbances such as drilling, blasting, and mechanical excavation inevitably induce dynamic impact loads, which lead to a significant initial damage level within the rock mass, manifesting as micropores and microcracks [2,3]. This initial damage level provides channels for groundwater infiltration, and this infiltration further weakens the mechanical properties of the rock mass, significantly increases the risk of geological disasters such as roadway instability and water inrush, and poses a serious threat to the safety of underground engineering [4,5].

At present, scholars have conducted extensive research on rocks with initial damage level, and freeze-thaw cycles [6–9] and heat treatment [10–14] are the most common methods for simulating this damage. Feng et al. [15] conducted indoor freeze-thaw cycle tests on yellow sandstone and found that as the number of freeze-thaw cycles increases, the porosity of the rock specimens increases significantly, while their uniaxial compressive strength decreases. Meng et al. [16] found that rock porosity increases as the number of freeze-thaw cycles increases, by simulating the deformation and failure processes of rocks with five different initial damage levels. Luo et al. [17] conducted biaxial compression tests on granites with different initial damage levels after high-temperature water cooling, revealing that the higher the initial damage level, the lower the rock’s biaxial strength and elastic modulus. Yang et al. [18] conducted uniaxial compression tests on granite subjected to different heat treatments, revealing that the crack damage and peak axial strain of the granite increased as the temperature rose. Although these studies provide an important reference for understanding the rock damage mechanism, they mainly focus on mechanical properties in static environments, whereas research on the initial damage level under dynamic impact loads is limited.

Additionally, the mechanical properties of rocks under water-rock interaction have attracted attention [19–23]. The mechanical behavior of rocks is not only controlled by their internal petrographic and structural characteristics, but also changes significantly with variations in moisture content [24]. In an experimental study, Khajevand [25] systematically quantified water’s weakening effect, revealing that the uniaxial compressive strength and Brazilian tensile strength of sedimentary rocks decreased by 18.97% and 24.95%, respectively, after water saturation. Zhou et al. [26] conducted dynamic mechanical tests on water-bearing sandstone, revealing that, at the same loading rate, the dynamic fracture initiation and propagation toughness of saturated specimens was significantly lower than that of dry specimens. Cheng et al. [27] conducted acoustic emission tests on sandstones with different soaking times under uniaxial compression, revealing that the presence of water caused the grain fracture mode to transition from transgranular fracture to intergranular fracture. Regarding evaluation methods, in addition to direct mechanical tests, the use of physical parameters such as P-wave velocity combined with soft computing methods (e.g., ANN and ANFIS) to predict the mechanical strength of sedimentary rocks has emerged as an important research frontier in recent years [28,29]. Most existing studies focus on the behavior of undamaged rocks under water-rock interaction, whereas research on rocks subjected to the coupling effect of the initial damage level and moisture content remains limited.

In summary, while many scholars have conducted extensive research on either the initial damage level in rock or water-rock interaction, research on the coupling effect of initial damage level and moisture content in deep rock mass engineering in water-rich conditions remains scarce. Therefore, in this study, we conduct the SHPB test, the XRD test, and the elastic longitudinal wave velocity test to analyze the dynamic mechanical properties, macroscopic failure patterns, fractal dimension, and energy dissipation patterns of the granite specimens under the coupling effect of varying initial damage levels and moisture content. Additionally, this study employs SEM technology to reveal the microscopic failure mode of granite specimens under the coupling effect of varying initial damage levels and moisture content. This study can provide a reference for the stability evaluation, disaster prevention, and control of deep rock mass engineering in water-rich conditions.

## 2 Materials and methods

**Fig 1:**
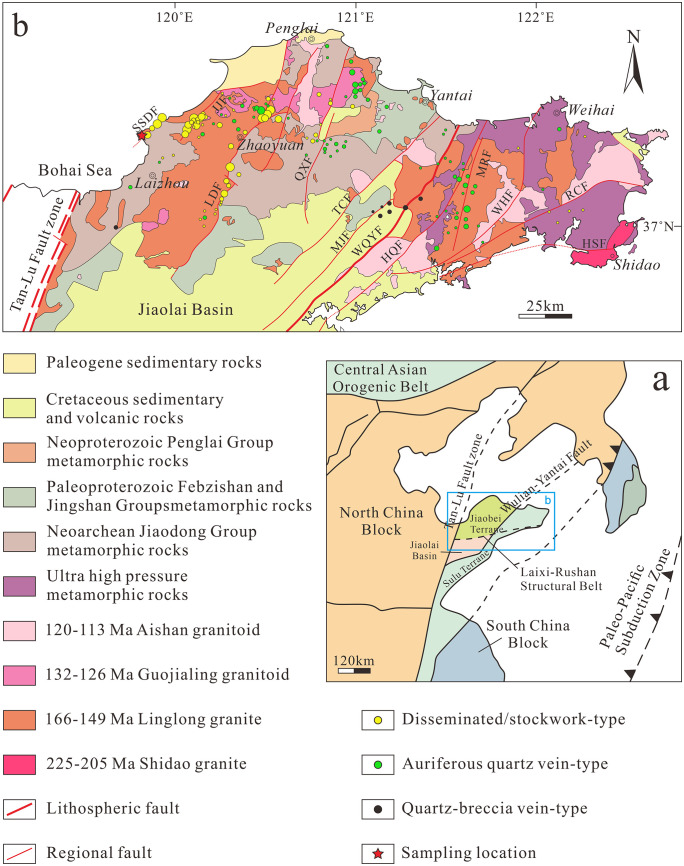
Geological map of the study area indicating sampling locations and the geological setting. The map was created by the authors based on geological data and tectonic framework adapted from [37] and [38]. Republished from [38] under a CC BY license, with permission from Yajie Feng, original copyright 2025.

Granite is a typical hard rock, which is widely distributed in various deep rock engineering projects, such as mines, tunnels, and underground caverns [30,31]. A mining area in eastern and northeastern Shandong Province, China, was selected as the research area. This mining area, located in the northwest of the Jiaodong Peninsula and bordered by the Bohai Sea on the west and north sides, belongs to the temperate monsoon climate zone. From a geological perspective, the mining area is part of the southeastern margin of the North China Craton and the Sulu Ultrahigh-Pressure Metamorphic Belt [32–34]. The complex geological structure of the Jiaodong area is controlled by a series of NE-trending (NNE–NE) major fault zones. From west to east, these fault zones are mainly the Sanshandao Fault, Jiaojia Fault, and Zhaoping Fault [35], with the study area being adjacent to the Sanshandao Fault Zone. The total extension length of this fault zone is approximately 20 km, with an onshore length of about 9.2 km. It trends NE overall, dips toward the SE, and has a dip angle ranging from 35° to 60°. The granite specimens used in this experiment were collected from the Linglong-type granite rock mass, deep within the study area. The granite specimen exhibits a porphyroid texture and a massive structure and contains quartz, which fills the space between other mineral particles in an anhedral granular form. Thus, the specimen possesses a uniform and dense texture. The mining area is situated deep within a coastal mine, so the rock mass has long been subjected to an environment of high ground stress and complex seawater seepage [36]. This provides a natural geological background for this study. Fig 1 presents the regional geological survey results and specimen collection locations of the study area.

### 2.1 Specimen preparation and mineral composition analysis

**Fig 2:**
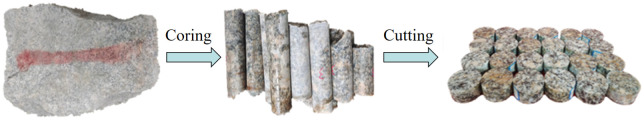
Granite specimen preparation.

To avoid any adverse impact on mechanical properties, the length to diameter ratio of the test specimens strictly adhered to the recommendations of the ISRM [39,40]. First, the specimens were inspected macroscopically, and only homogeneous, isotropic, unweathered rocks free of visible joints, cracks, fissures, or other discontinuities were selected. Then, the selected rocks were cut and polished to fabricate cylindrical specimens with a diameter of 50 mm and a height of 25 mm. The roughness of the end faces was ≤ 0.02 mm, and the axial perpendicularity was ≤ 0.25°. Representative prepared granite specimens are shown in Fig 2. The physical and mechanical properties of the granite specimens were determined as follows: longitudinal wave velocity was measured with a ZT801 geotechnical acoustic tester; uniaxial compressive strength (UCS) was obtained using an RMT-150C rock mechanics test system; and mass was recorded with a high-precision electronic balance. The basic physical and mechanical parameters of the granite are summarized in Table 1.

**Table 1:** Basic physical and mechanical parameters of the granite.

Diameter(mm)	Height(mm)	Mass(g)	Density (g/cm³)	P-wave velocity (m/s)	Uniaxial compressive strength(MPa)	Elastic modulus(GPa)
50	25	127.63	2.61	5682	145.2	24.56

**Fig 3:**
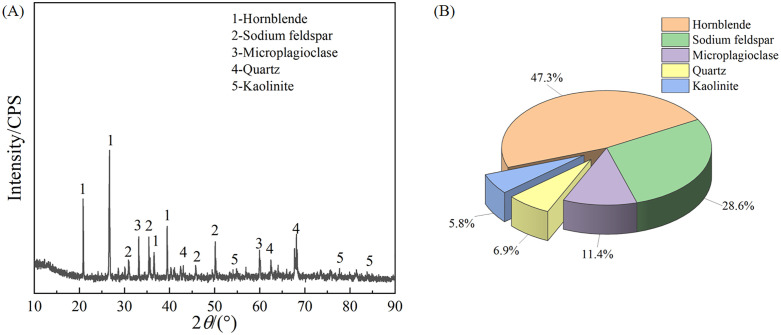
Mineralogical analysis of the granite specimen. (A) XRD pattern. (B) Mineral composition by mass fraction.

A rock’s sensitivity to both its initial damage level and water-saturated state is largely governed by its mineralogy [41]. Therefore, XRD was employed to determine the granite’s mineral composition and its corresponding mass fractions. The XRD results (Fig 3) indicate that the granite is primarily composed of hornblende (47.3%), feldspar (40.0%), quartz (6.9%), and kaolinite (5.8%). The feldspar component comprises sodium feldspar (28.6%) and microplagioclase (11.4%).

### 2.2 Design of initial damage levels and moisture states of specimens

**Fig 4:**
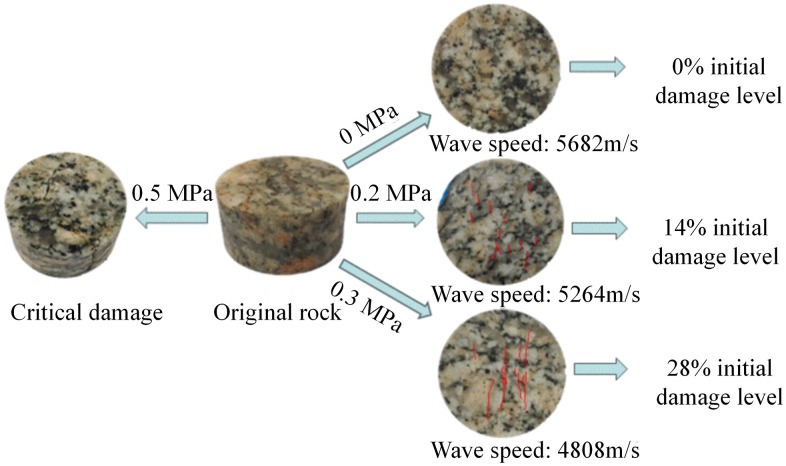
Granite specimens with varying initial damage levels.

The initial step in preparing granite specimens with graduated levels of damage was to identify the threshold air pressure for damage initiation through preliminary SHPB tests. This threshold pressure was determined to be 0.5 MPa. Therefore, two sub-critical impact air pressures, 0.2 MPa and 0.3 MPa, were selected to induce different initial damage levels in the granite specimens. The pre-damaging process yielded two sets of specimens with average P-wave velocities of 5264 m/s and 4808 m/s, respectively. The initial damage level is quantified using the damage variable *D*_n_, which is calculated via the formula *D*_n_ = 1−(*v*/*v*_0_)^2^ based on the change in P-wave velocity. Here, *v*_0_ denotes the P-wave velocity of the specimen before pre-damage, and *v* represents the P-wave velocity of the specimen after pre-damage [42–44]. Based on this, the initial damage levels for the two pre-damaged groups were calculated to be 14% and 28%, respectively. The unimpacted specimens served as the control group (0% initial damage level). Representative granite specimens from each initial damage level are shown in Fig 4.

To investigate the influence of moisture content on pre-damaged granite, specimens were prepared with four distinct moisture states [20,45]. Specimens with varying initial damage levels were tested under four distinct moisture states: dry state, natural moisture state, natural saturated state, and forced saturated state. For the dry state, specimens were dried in an oven at 105 °C for 24 hours. Subsequently, their masses were measured and recorded as *m*_d_, and they were immediately sealed with plastic film to prevent moisture absorption from the air. For the natural moisture state, dry specimens were placed on a stand in an evaporating dish. Water was then slowly introduced to immerse the bottom of the specimens, after which they were allowed to stand for 72 hours. For the natural saturated state, dry specimens were placed in a container. Water was then incrementally added to reach 1/4, 1/2, and 3/4 of the specimen’s height, with each immersion level allowed to stand for 2, 4, and 6 hours, respectively. Finally, water was added to a level 2–3 cm above the specimen’s top surface, followed by complete immersion for 48 hours. For the forced saturated state, the naturally saturated specimens were heated in a boiling chamber for 6 hours to ensure complete expulsion of air from their pores. After each water treatment, the specimens were removed and their surfaces wiped dry. Each specimen’s mass (*m*_a_) was then recorded, and it was immediately wrapped in plastic film to prevent moisture exchange with the environment. The moisture content (*W*) for each moisture state was determined using the gravimetric method, as defined by Equation (1):


$$W=\frac{m_{a}-m_{d}}{m_{d}}\times 100\%$$ (1)

where *W* is the moisture content, *m*_a_ is the mass of the specimen after water absorption, and *m*_d_ is the mass of the dry specimen.Table 2 presents the physical parameters of granite specimens corresponding to varying moisture states. As shown in Table 2, the moisture content of the specimens increases progressively across the dry state, natural moisture state, natural saturated state, and forced saturated state.

**Table 2:** Physical parameters of granite specimens under varying initial damage levels and moisture states.

No.	Initial damage level(%)	Moisture state	Diameter(mm)	Height (mm)	Moisture Content(%)
1−1	0	Dry state	50.02	25.06	0
1-2		Natural moisture state	49.96	25.04	0.093
1-3		Natural saturated state	50.02	25.08	0.217
1-4		Forced saturated state	49.98	25.02	0.288
2−1	14	Dry state	49.98	25.08	0
2−2		Natural moisture state	49.96	25.02	0.095
2-3		Natural saturated state	50.04	24.98	0.244
2-4		Forced saturated state	50	25.1	0.295
3−1	28	Dry state	50.08	25.02	0
3−2		Natural moisture state	49.98	25.04	0.098
3−3		Natural saturated state	50.1	25.1	0.271
3-4		Forced saturated state	50.06	25	0.307

### 2.3 Experimental Setup and Procedure

**Fig 5:**
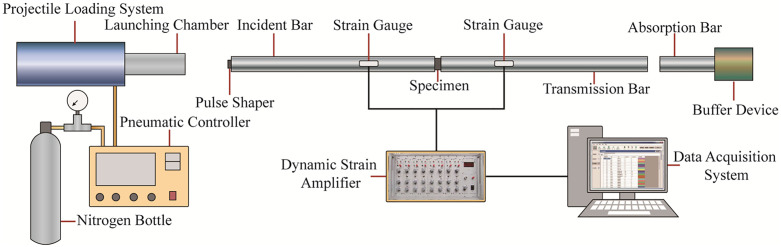
Schematic of the SHPB system.

The SHPB system is composed of three main subsystems: a pressure bar assembly, a projectile loading system, and a data acquisition system. The pressure bar assembly is composed of three main components: the incident bar, the transmission bar, and the absorption bar. All bars are composed of the same material, possessing a modulus of elasticity of 210 GPa and a stress wave velocity of 5190 m/s. The projectile loading system uses a 400-mm-long, 37-mm-diameter steel projectile driven by high-pressure nitrogen gas. The impact velocity is precisely regulated by a pneumatic controller. The data acquisition system comprises a dynamic strain amplifier and a high-speed oscilloscope. Operating at a 10 MHz sampling frequency, the system is capable of precisely recording the stress-strain behavior that occurs during dynamic deformation. The SHPB system is shown in Fig 5.

**Fig 6:**
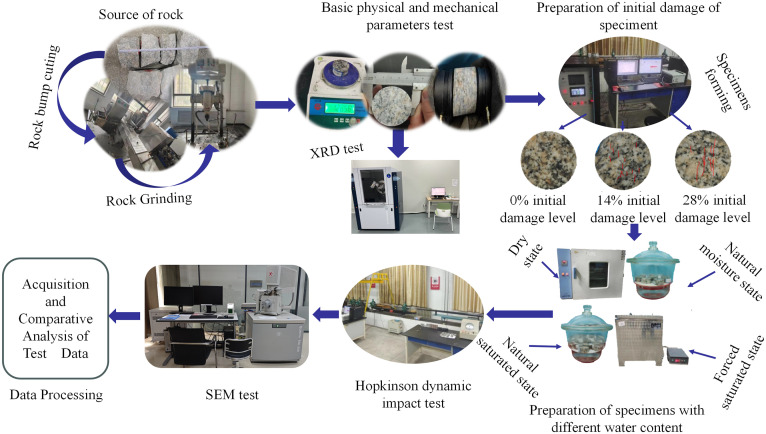
Overall testing flowchart.

To avoid oscillation of the stress waveform and satisfy the assumption of one-dimensional elastic stress waves, a square brass plate, 15 mm in side length and 0.3 mm in thickness, was pasted at the center of the incident end face to serve as a pulse shaper. Additionally, Vaseline was spread at the contact positions between the specimens and the end faces of the incident and transmission bars to reduce friction at the specimen ends, thereby minimizing the end effect [46,47]. All tests were conducted at the same room temperature. First, an impact test without a specimen must be conducted on the SHPB system before installing the specimen to check the proper connection of the bars, the transmission of the stress wave, and the working condition of the data acquisition system. After the test system’s performance meets the test requirements, the specimen is installed between the incident bar and the transmission bar, and the incident bar is pushed to the same position in each test [48,49]. Then, after the installation of the specimen is completed, and with the nitrogen bottle and pneumatic controller confirmed to be closed, a plastic soft rod is used to push the projectile to the preset position, and a mark is made on the soft rod. In subsequent tests, the preset position is determined by this mark to ensure the consistency of the velocity when the projectile impacts the incident bar. Then, when the projectile is returned to the preset position, the pneumatic controller is opened, the preset pressure value of 0.5 MPa is inputted, and the impact pressure is adjusted by presetting the air pressure value in the nitrogen bottle. Then, the pressure valve of the nitrogen bottle is opened to start the air intake, and the pressure valve is closed after the inflation is completed to wait for the impact. Then, the start button is clicked, the projectile is fired, and the test begins. After the final impact, the air pressure valve of the Nitrogen Bottle is closed, and the test results are observed. To ensure the reliability and representativeness of the test results, at least three replicate tests were conducted for each group of testing conditions (i.e., a combination of each initial damage level and moisture state). In this study, the representative test results for each group of testing conditions were selected for analysis. The entire test process is illustrated in Fig 6.

## 3 Results

### 3.1 Dynamic stress-strain behavior

**Fig 7:**
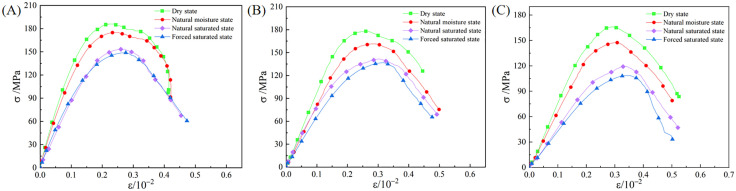
Influence of initial damage level and moisture state on the stress-strain behavior of granite. (A) 0% Initial damage level. (B) 14%Initial damage level. (C) 28% Initial damage level.

Typical dynamic stress-strain curves of granite under varying initial damage levels and moisture states are presented in Fig 7. The stress-strain curves depicted in the figure generally exhibit four characteristic phases: the initial compaction phase, the quasi-elastic deformation phase, the elastoplastic deformation phase, and the post-peak failure phase. The initial loading stage is characterized by distinct compaction behavior, which is particularly prominent in moisture-containing specimens and those with existing damage. The upwardly concave shape of the curves is a manifestation of the compaction of initial pores and microcracks within the specimens during impact loading. As the stress level increases further, the curve enters the quasi-elastic deformation stage. In this stage, the curve is approximately linear, and its slope represents the specimen’s dynamic modulus of elasticity. As the stress continues to increase, the curve enters the elastoplastic deformation stage, during which cracks continuously initiate and propagate. This results in a gradual decrease in the curve’s slope, indicating the onset of plastic deformation. The progressive extension and linkage of macroscopic cracks occurs within the specimen as the stress approaches the dynamic peak strength. Consequently, the specimen’s load-bearing capacity rapidly diminishes, and the stress-strain curve transitions into the post-peak failure regime. This process is characteristic of the material’s failure behavior under impact loading.

The data in Fig 7 reveal an inverse relationship between dynamic peak stress and peak strain when the initial damage level is held constant. Specifically, as the moisture content is increased from a dry to a forced saturated state, the dynamic peak stress progressively declines while the peak strain concurrently rises. For example, for specimens with 0% initial damage level (Fig 7A), the dynamic peak stress decreased from 186.1 MPa (dry state) to 148.8 MPa (forced saturated state), representing a reduction of 20.0%. Conversely, the peak strain for these specimens increased from 0.23 × 10^−2^ to 0.27 × 10^−2^, representing an increase of 17.4%. When the moisture state was held constant, the dynamic peak stress and peak strain exhibited an inverse response to an increase in the initial damage level (from 0% to 28%). Specifically, the dynamic peak stress was observed to decrease while the peak strain increased. For example, for dry specimens, the dynamic peak stress decreased from 186.1 MPa at 0% initial damage level to 166.1 MPa at 28% initial damage level, representing a reduction of 10.7%. Conversely, the peak strain for these specimens increased from 0.23 × 10^−2^ to 0.31 × 10^−2^, representing an increase of 34.8%.

### 3.2 Variation of dynamic peak stress

**Fig 8:**
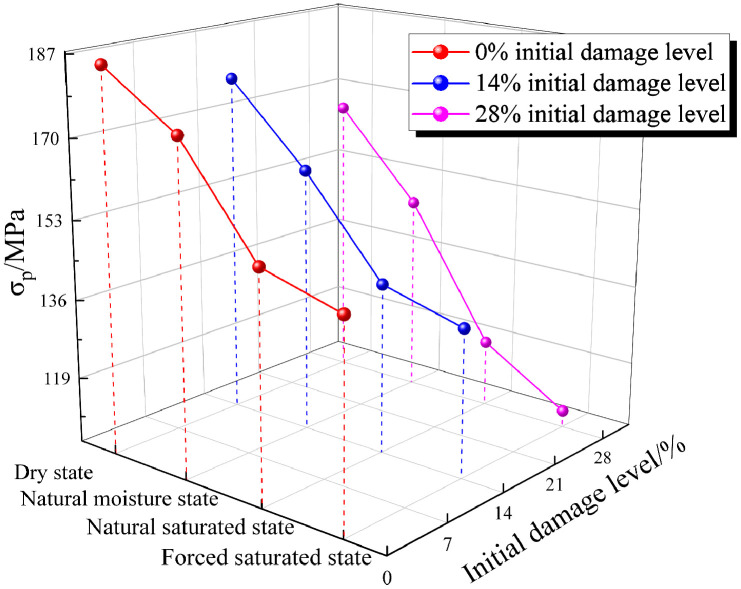
Dynamic peak stress variation.

Dynamic peak stress is defined as the maximum stress a specimen can withstand under dynamic compression, and it is a key parameter used to assess a material’s resistance to rapid crack propagation and damage [50,51]. The variation of dynamic peak stress in granite with varying initial damage levels and moisture states is illustrated in Fig 8. Holding the moisture state constant, an increase in the initial damage level exerts a clear weakening effect, causing a monotonic decrease in the dynamic peak stress (Fig 8). Conversely, at a given initial damage level, an increase in moisture content leads to a decrease in dynamic peak stress. For specimens with 0% initial damage level, the dynamic peak stress decreased from 186.1 MPa (dry state) to 175.2 MPa (natural moisture state), 153.5 MPa (natural saturated state), and 148.8 MPa (forced saturated state), representing decreases of 5.9%, 17.5%, and 20.0%, respectively. For specimens with a 14% initial damage level, the dynamic peak stress decreased from 178.1 MPa to 161.6 MPa, 141.4 MPa, and 136.7 MPa as the moisture state transitioned, representing reductions of 9.3%, 20.6%, and 23.2%, respectively. For specimens with a 28% initial damage level, the dynamic peak stress decreased from 166.1 MPa to 147.5 MPa, 119.4 MPa, and 108.6 MPa as the moisture state transitioned, representing reductions of 11.2%, 28.1%, and 34.6%, respectively. Under the 28% initial damage level and forced saturated state, the dynamic peak stress of the specimen decreased by 41.6% compared to that under the 0% initial damage level and dry state. The decrease in dynamic peak stress is attributed to the weakening of the cohesive force between rock particles after water molecules enter the rock’s pores and fissures. Additionally, the presence of water may promote the expansion and coalescence of microcracks in the rock, thereby reducing the rock’s strength.

In the dry state, as the initial damage level increased from 0% to 14% and 28%, the dynamic peak stress decreased from 186.1 MPa to 178.1 MPa and 166.1 MPa, respectively, representing decreases of 4.3% and 10.7%. In the natural moisture state, as the initial damage level increased, the dynamic peak stress decreased from 175.2 MPa to 161.6 MPa and 147.5 MPa, representing decreases of 7.8% and 15.8%, respectively. In the natural saturated state, as the initial damage level increased, the dynamic peak stress decreased from 153.5 MPa to 141.4 MPa and 119.4 MPa, representing decreases of 7.9% and 22.2%, respectively. In the forced saturated state, as the initial damage level increased, the dynamic peak stress decreased from 148.8 MPa to 136.7 MPa and 108.6 MPa, representing decreases of 8.1% and 27.0%, respectively. This suggests that the combined effects of initial damage level and moisture content significantly exacerbate the deterioration of material strength.

To further quantify the weakening of the specimen’s dynamic peak stress caused by initial damage level and moisture state, a water-damage weakening coefficient *f*_n_ is introduced, as shown in Equation (2) [52]:


$$f_{n}=\text{1}-\frac{\sigma_{s}}{\sigma_{d}}$$ (2)

where $\sigma_{s}$ is the dynamic peak stress of the specimen under varying initial damage levels and moisture states, and $\sigma_{\text{d}}$ is the dynamic peak stress of the undamaged specimen in the dry state. A larger *f*_n_ value indicates a more pronounced weakening of the specimen’s dynamic peak stress due to the coupled effect of initial damage level and moisture content. Table 3 presents the dynamic peak stress weakening coefficients for specimens under varying initial damage levels and moisture states.

**Table 3:** Dynamic peak stress weakening coefficients for specimens with varying initial damage levels.

Initial damage level(%)	Moisture state	Dynamic peak stress(MPa)	Water-damage weakening coefficient
0	Dry	186.1	0
	Natural moisture state	175.2	0.06
	Natural saturated state	153.5	0.18
	Forced saturated state	148.8	0.20
14	Dry	178.1	0.04
	Natural moisture state	161.6	0.13
	Natural saturated state	141.4	0.24
	Forced saturated state	136.7	0.27
28	Dry	166.1	0.11
	Natural moisture state	147.5	0.21
	Natural saturated state	119.4	0.36
	Forced saturated state	108.6	0.42

**Fig 9:**
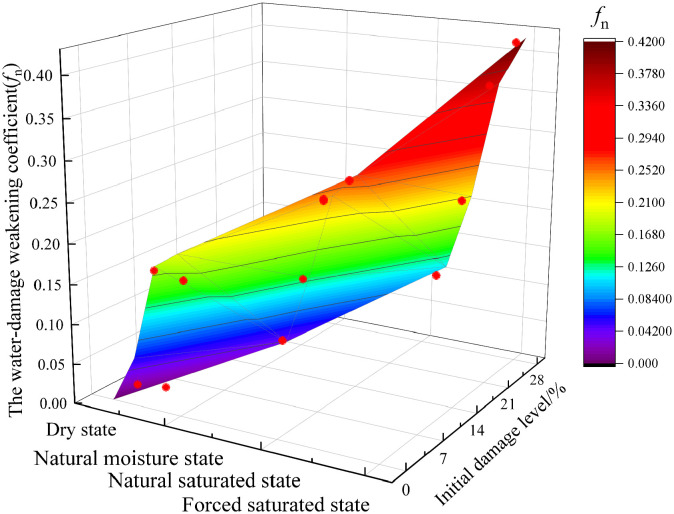
Change in water-damage weakening coefficient (*f*_n_).

Fig 9 illustrates the variation patterns of the dynamic peak stress water-damage weakening coefficient for granite specimens under varying initial damage levels and moisture states. As shown in the figure, the water-damage weakening coefficient (*f*_n_) increases with both the initial damage level and moisture content (i.e., as the moisture state transitions from the dry state to the forced saturated state). Furthermore, a higher initial damage level leads to a more pronounced weakening effect of moisture content on the dynamic mechanical properties of granite specimens. Specifically, for granite specimens, the water-damage weakening coefficient (*f*_n_) increased as the moisture content transitioned from dry to forced saturated state. For the 0% initial damage level, *f*_n_ increased from 0 to 0.20. For the 14% initial damage level, *f*_n_ increased from 0.04 to 0.27. For the 28% initial damage level, *f*_n_ increased from 0.11 to 0.42.

### 3.3 Variation of peak modulus

For a nonlinear stress-strain curve, the stiffness of a material is typically described by two types of moduli: the tangent modulus and the secant modulus. In dynamic impact tests, the peak modulus provides a simpler and more convenient approach for calculating the dynamic elastic modulus compared to the conventional tangent modulus. Furthermore, it avoids the curve-fitting uncertainties associated with determining the secant modulus. Therefore, the peak modulus was adopted in this study to represent the dynamic elastic modulus, as it directly reflects the rock’s resistance to deformation under impact loading [53].

**Fig 10:**
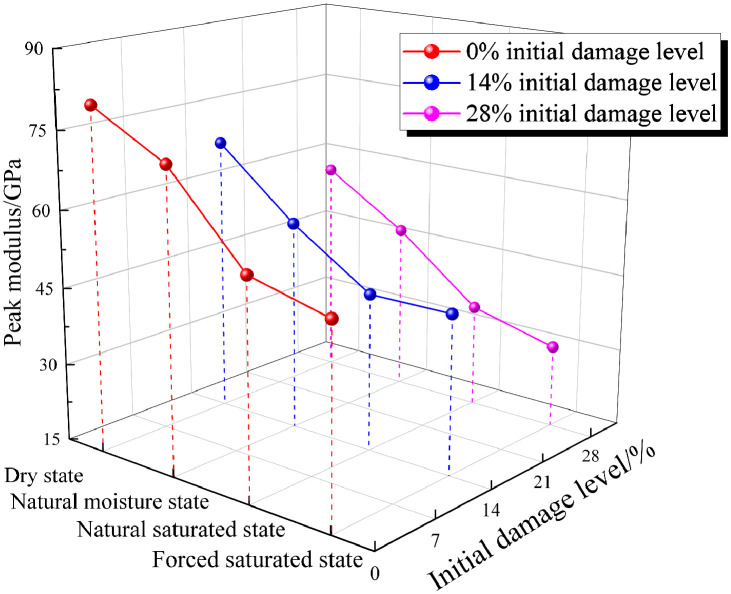
Dynamic peak modulus variation.

The variation of the peak modulus with initial damage level and moisture state is illustrated in Fig 10. The peak modulus of the granite specimens generally decreased with an increase in either the initial damage level or the moisture content. Specifically, for specimens with 0% initial damage level in the dry state, the peak modulus was 80.87 GPa. As moisture content increased, the peak modulus decreased to 73 GPa, 56.85 GPa, and 53.14 GPa, representing decreases of 9.7%, 29.7%, and 34.3%, respectively. When specimens were in the dry state, the peak modulus decreased from 80.87 GPa to 68.5 GPa and 57.28 GPa as the initial damage level increased from 0% to 14% and 28%, representing decreases of 15.3% and 29.2%, respectively. Under the 28% initial damage level and forced saturated state, the peak modulus of the specimen decreased by 61.6% compared to that under the 0% initial damage level and dry state. This trend is consistent with the relationship *E*_eff_=(1 − *D*_k_)*E*, which describes the effective elastic modulus and damage factor in damage mechanics [42].

To quantify the coupled deterioration effect of initial damage level and moisture content, a peak modulus weakening coefficient (*f*_k_) for the specimen is introduced, analogous to the water-damage weakening coefficient in Equation (2). The results, summarized in Table 4, indicate that the deterioration of the specimens under the coupled effect of initial damage level and moisture content is significantly greater than that caused by either factor acting in isolation. Specifically, the peak modulus weakening coefficient (*f*_k_) for granite specimens varies as follows: When the specimen is a dry state and has a 28% initial damage level, its *f*_k_ is 0.29. When the specimen is in a forced saturated state and has 0% initial damage level, its *f*_k_ is 0.34. When the specimen is in a forced saturated state and has a 28% initial damage level, its *f*_k_ is 0.62. These results clearly indicate that granite specimens subjected to the coupled effect of both factors exhibit the most significant deterioration.

**Table 4:** Peak modulus weakening coefficients for specimens with varying moisture states and initial damage levels.

Peak modulus weakening coefficients				
**Initial damage level (%)**	**Dry**	**Natural moisture state**	**Natural saturated state**	**Forced saturated state**
0	0	0.10	0.30	0.34
14%	0.15	0.31	0.44	0.43
28%	0.29	0.41	0.57	0.62

### 3.4 Macroscopic failure patterns

**Fig 11:**
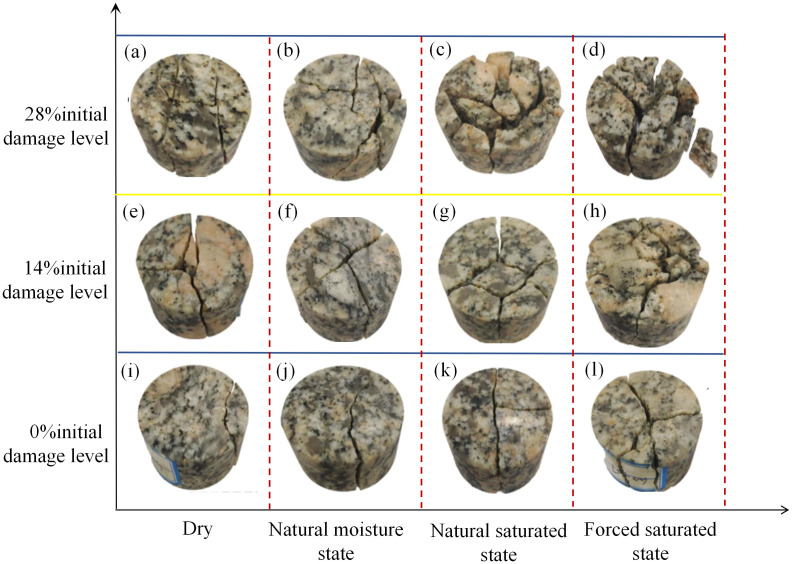
Macroscopic failure patterns of specimens under varying initial damage levels and moisture states.

Typical macroscopic failure patterns of selected granite specimens under varying initial damage levels and moisture states are illustrated in Fig 11. As shown in Fig 11I, the undamaged specimen in the dry state exhibited typical axial splitting, a form of brittle failure. This failure was characterized by a dominant axial crack that fractured the specimen into a few large, irregular fragments. With an increase in either the initial damage level or moisture content, the failure mode transitioned from simple tensile or shear fractures to a more complex mixed-mode failure involving both tension and shear (Fig 11D). Specifically, at a constant initial damage level, an increase in moisture content significantly intensifies the degree of fragmentation. This is evidenced by an increase in the number of smaller fragments. The failure morphology also evolves from simple brittle fracture to a more complex, mixed-mode fracture. When the moisture state is unchanged, an increasing initial damage level leads to finer and more irregular crushed fragments. Notably, for specimens with a 28% initial damage level in the forced saturated state, the final morphology exhibits holistic failure, indicating a loss of structural integrity. This phenomenon indicates that the coupled effect of initial damage level and moisture content has a synergistic, deteriorating influence on the dynamic integrity of granite specimens. This phenomenon indicates that initial damage level and moisture content act synergistically to degrade the dynamic integrity of the granite specimens.

While the failure characteristics and fragment size after rock damage can intuitively describe the extent of rock specimen damage, this qualitative approach is often imprecise. To quantify the complexity of impact-induced surface fracture networks in granite specimens, this study introduces the box-counting fractal dimension based on image analysis [54]. The corresponding formula is presented in Equation (3).


$$N\left(\epsilon \right)\propto \epsilon^{-D}$$ (3)

By taking the logarithm of both sides of Equation (3), it can be transformed into a linear relationship, thereby providing a rigorous mathematical definition of the fractal dimension *D*, as shown in Equation (4).


$$D=-\underset{\epsilon \to 0}{lim}\frac{log(N(\epsilon ))}{log\left(\epsilon \right)}$$ (4)

where *D* is the fractal dimension of the cracks on the specimen’s surface; ϵ is the side length of the square grid boxes used to cover the image, which represents the spatial scale of the measurement; and N(ϵ) is the total number of boxes intersecting the crack network at scale ϵ.

The box-counting fractal dimension is used to quantify the cracks on the specimen’s surface. This involves first grayscaling and binarizing the surface image of the damaged specimen to extract the geometric form of its crack network. Then, the crack network was covered by a series of square grids with varying side lengths (ϵ). For each scale, the number of effective grids (N(ϵ)) intersecting the cracks was counted. Finally, the fractal dimension *D* is calculated using the formula. A larger *D* value indicates a more diffuse crack distribution and a more complex morphology. The trend of this change directly reflects the influence of varying initial damage levels and moisture states on the specimen’s failure mode.

**Fig 12:**
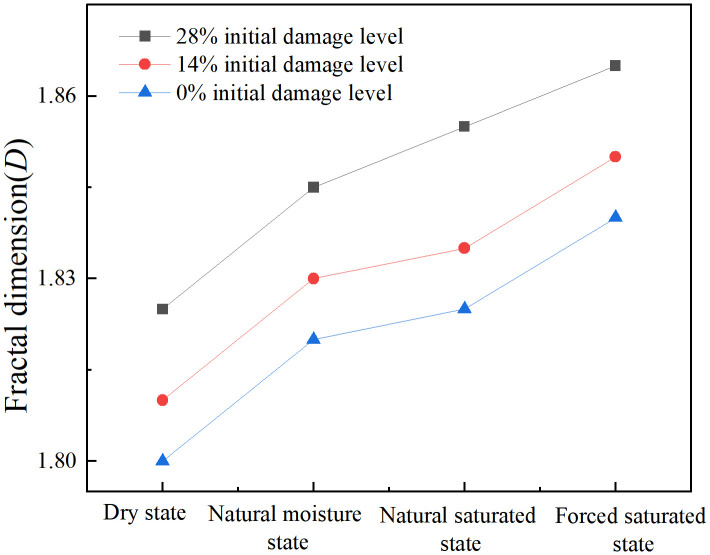
Fractal dimension curves of specimens with varying initial damage levels and moisture states.

Fig 12 presents the fractal dimension curve of specimens under varying initial damage levels and moisture states. As shown in Fig 12, the fractal dimension (*D*) exhibits a monotonically increasing trend with both the initial damage level and moisture content. When the moisture content of the specimen is constant, a more severe initial damage level leads to a larger fractal dimension. For example, in the dry state, the fractal dimension increases from 1.801 (at 0% initial damage level) to 1.824 (at 28% initial damage level). Conversely, when the initial damage level of the specimen is constant, a higher moisture content results in a larger fractal dimension. For example, at the 28% initial damage level, the fractal dimension increases from 1.824 (in the dry state) to 1.865 (in the forced saturated state). The fractal dimension of specimen fragmentation reached a maximum of 1.865 at the 28% initial damage level and in the forced saturated state, while a minimum of 1.801 was observed at the 0% initial damage level in the dry state. This quantitative result aligns with the previous conclusions regarding the macroscopic fragmentation patterns of the specimens. Specifically, it confirms that the degree of failure of the specimens is exacerbated by the coupled effect of initial damage level and moisture state.

### 3.5 Energy dissipation density analysis

Defects within the granite, resulting from initial damage level and moisture content treatments, attenuate stress wave propagation. Both the dynamic peak stress and the energy evolution of the damaged granite under impact loading are influenced by this phenomenon. The incident energy (*W*_I_), reflected energy (*W*_R_), and transmitted energy (*W*_T_) of the granite specimen under impact loading can be calculated from Equation (5) to (7) based on one-dimensional wave theory and energy conservation principles [55–57]. The study shows that most of the energy absorbed by the granite is consumed in crushing. Ignoring the energy consumption between the granite and the compression bar, the absorbed energy (*W*_S_) can be calculated from Equation (8) based on the principle of energy dissipation. Additionally, to minimize errors caused by variations in specimen size, the energy dissipated per unit volume during granite crushing, i.e., the energy dissipation density (*w*_s_), was introduced, as shown in Equation (9) [58–60].


$$W_{\text{1}}\left(t\right)=AEC_{\text{0}}^{\tau }\int_{\text{0}}^{t}\varepsilon_{\text{1}}^{\text{2}}\left(t\right)\text{d}t$$ (5)

$$W_{R}\left(t\right)=AEC_{\text{0}}^{\tau }\int_{\text{0}}^{t}\epsilon_{R}^{\text{2}}\left(t\right)\text{d}t$$ (6)

$$W_{T}\left(t\right)=AEC_{\text{0}}^{\tau }\int_{\text{0}}^{t}\varepsilon_{T}^{\text{2}}\left(t\right)\text{d}t$$ (7)

$$W_{S}\left(t\right)=W_{I}\left(t\right)-W_{R}\left(t\right)-W_{T}\left(t\right)$$ (8)

$$\omega_{s}=\frac{W_{s}}{V_{s}}=\frac{\left(W_{I}-W_{R}-W_{T}\right)}{V_{s}}$$ (9)

where $\varepsilon_{\text{1}}^{\text{2}}\left(t\right)$, $\varepsilon_{R}^{\text{2}}\left(t\right)$, and $\varepsilon_{T}^{\text{2}}\left(t\right)$ are the incident, reflected, and transmitted waves on the pressure bar at time *t*, respectively; $C_{\text{0}}^{\tau }$ is the longitudinal wave velocity of the pressure bar; and *V*_S_ denotes the volume of the granite specimen.

**Fig 13:**
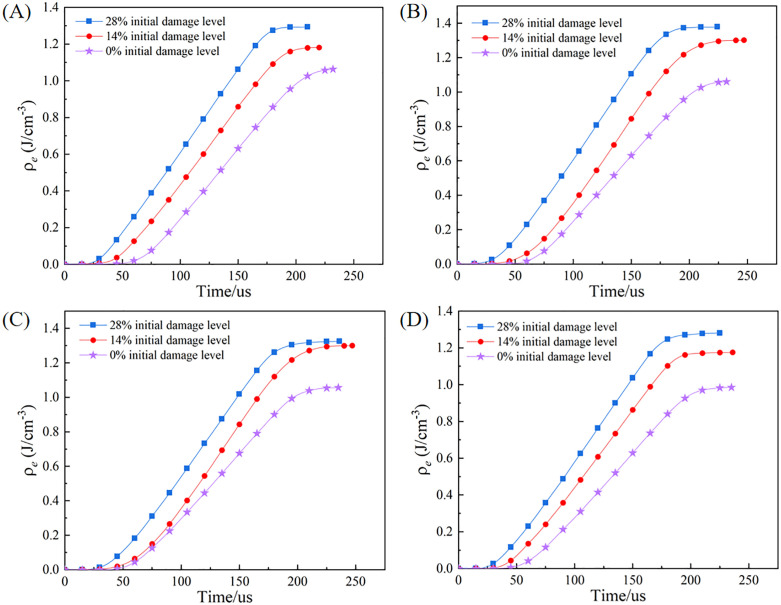
Energy dissipation density of specimens under varying initial damage levels and moisture states. (A) Dry state. (B) Natural moisture state. (C) Natural saturated state. (D) Forced saturated state.

Fig 13 plots the energy dissipation density as a function of both initial damage level and moisture state. A significant increase in the energy dissipation density is observed with an elevation of the initial damage level when the moisture state is held constant, a trend clearly shown in the figure. The undamaged (0% initial damage level) specimen, when tested under dry conditions, exhibited an energy dissipation density of 1.063 J/cm^3^. For specimens with 14% and 28% initial damage levels, this energy dissipation density increases to 1.184 J/cm^3^ and 1.295 J/cm^3^, respectively, representing increases of 11.4% and 21.8% (compared to the undamaged specimen in the dry state). The underlying mechanism is that a higher initial damage level implies a greater number of pre-existing microcracks and discontinuous interfaces. Under dynamic impact, frictional slip along these interfaces and the propagation of a more complex crack network both dissipate a larger amount of energy. At a constant initial damage level, the energy dissipation density first increases and then decreases as the moisture content rises, peaking in the natural moisture state. For example, for specimens with a 14% initial damage level, the energy dissipation density increased from 1.184 J/cm^3^ in the dry state to 1.299 J/cm^3^ in the natural moisture state, representing an increase of 9.7%. However, it then decreased to 1.175 J/cm^3^ in the forced saturated state, representing a 9.5% reduction relative to the natural moisture state. This phenomenon can be explained by the dual role of moisture in the rock’s failure process. On one hand, a small amount of moisture can increase the fracture toughness at microcrack tips, requiring more energy for crack propagation. On the other hand, upon saturation, the lubricating and hydro-wedging effects of water become dominant, significantly lowering the rock’s damage threshold and thus reducing the total dissipated energy.

### 3.6 Microscopic failure modes

**Fig 14:**
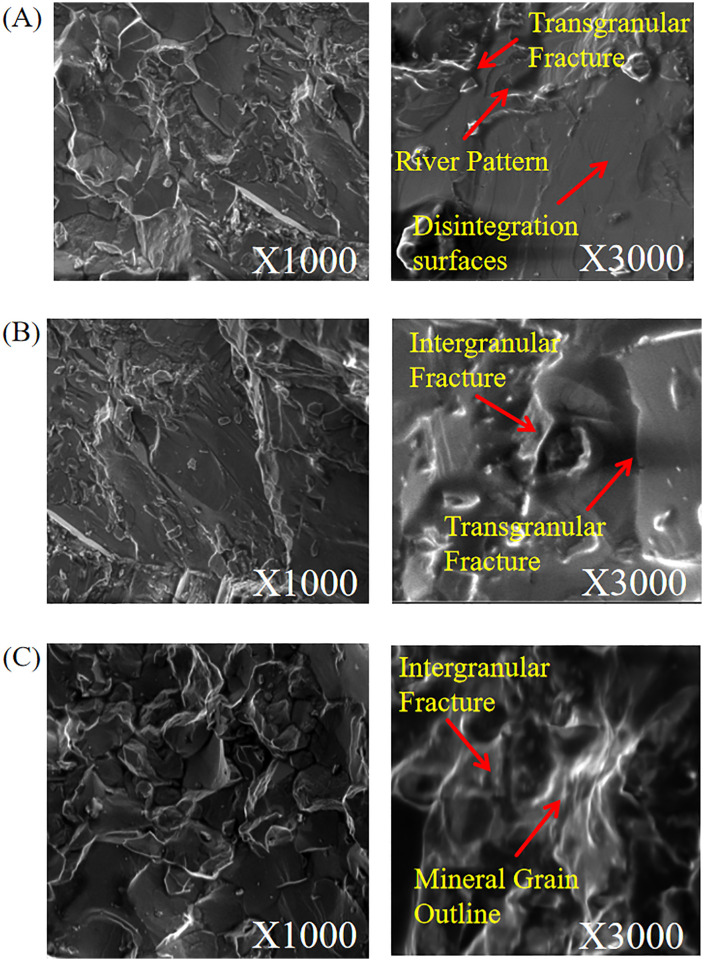
Typical microscopic failure mode of specimens with varying initial damage levels and moisture states. (A) 0% Initial damage level in the dry state. (B) 14% Initial damage level in the natural saturated state.(C) 28% Initial damage level and forced saturated state.

To further elucidate the microscopic failure modes resulting from the influence of initial damage level and moisture content on the dynamic failure of granite specimens, SEM observations were conducted on typical specimen fractures, as shown in Fig 14. As the initial damage level and moisture content increase, the microscopic failure mode of the granite specimens transitions from predominantly transgranular to intergranular fracture, as illustrated in the figure. At the 0% initial damage level and in the dry state, the specimen fracture exhibited typical transgranular characteristics (Fig 14A). Its macroscopic fracture surface is relatively flat, with large, smooth cleavage planes and step-like river patterns visible upon magnification. At the 14% initial damage level and in the natural moisture state, the specimen’s fracture exhibited a mixed mode of transgranular and intergranular failure (Fig 14B). The fracture morphology became more complex, characterized by both flat transgranular cleavage planes and numerous uneven intergranular damage zones resulting from grain separation. At the 28% initial damage level and under the forced saturated state, the specimen’s fracture exhibits typical characteristics of predominant intergranular failure (Fig 14C). Its fracture surface is extremely rough and loose, revealing clear outlines of mineral grains and numerous intergranular microcracks under high magnification.

In summary, SEM microscopic observations clearly reveal that the dynamic microscopic failure mode of granite varies with both the initial damage level and moisture state. Specifically, the mechanism gradually transitions from transgranular fracture (at the 0% initial damage level in the dry state) to a mixed mode of transgranular and intergranular fracture (at the 14% initial damage level in the natural saturated state). It ultimately evolves to predominantly intergranular fracture (at the 28% initial damage level in the forced saturated state). This indicates that the failure mode is dominated by intergranular fracture under conditions of the 28% initial damage level in the forced saturated state. The intrinsic mechanism of this evolutionary process lies in the continuous weakening of mineral grain bonding strength due to the coupled effect of the initial damage level and moisture. This allows grain boundaries to progressively replace the crystals themselves as the preferred channels for crack propagation and the lowest impedance paths for energy release. This transition from high-energy transgranular fracture to low-energy intergranular fracture is consistent with the weakening of macroscopic mechanical properties observed previously.

## 4 Discussion

In deep geotechnical engineering, the dynamic load acting on surrounding rock due to construction disturbance induces initial damage levels, and the surrounding rock is often in a water-rich environment. Therefore, its structure is prone to instability. Feng et al. [15] and Meng et al. [16] studied the rock damage mechanism by simulating the initial damage level via the freeze-thaw cycle method, and found that an increase in the number of freeze-thaw cycles significantly increases rock porosity and reduces its mechanical properties. Although these studies provide an important reference for understanding the rock damage mechanism, they mainly focus on the mechanical properties of rock under static load, and there is limited research on the rock initial damage level under dynamic load.

In this study, different initial damage levels induced by dynamic load are simulated via the SHPB test, and granite specimens are subjected to varying moisture content treatments to explore their dynamic mechanical properties and energy dissipation patterns. First, we tested the specimens with the same initial damage level but varying moisture contents, revealing that water has a weakening effect on the mechanical properties of rock. This is consistent with the results of Yin et al. [20] and Khajevand [25]. However, this study proposes a water-damage weakening coefficient *f*_n_, which further quantifies the water weakening effect. For example, when the initial damage level is 28%, the water-damage weakening coefficient *f*_n_ increases from 0.11 to 0.42, and the dynamic peak stress of the specimen decreases by 34.6% as the moisture state changes from dry to forced saturated. At the same time, unlike previous studies, this study finds that the initial damage level of the specimens has an amplification effect on the water weakening effect. For example, in the natural saturation state, as the initial damage level increases from 0% to 14% and 28%, the water-damage weakening coefficient *f*_n_ increases from 0.18 to 0.24 and 0.36, and the dynamic peak stress of the specimen decreases by 7.9% and 22.2%, respectively. Second, this study finds that the energy dissipation density of the specimen first increases and then decreases as the moisture content increases. This is different from the research results of Zhou et al. [26], which found that the dynamic fracture toughness of sandstone decreases significantly under the water-saturated state, but no nonlinear pattern in energy dissipation density was observed. This indicates that there may be significant differences in the energy dissipation density behavior of different rock types under water-rock interaction. Finally, through the analysis of SEM test results, this study finds that as the initial damage level and moisture content increase, the microscopic failure mode of the specimen transitions from transgranular fracture to mixed-mode fracture, and finally to intergranular fracture. This microscopic failure mode is consistent with the conclusion of Luo et al. [17], who found that the macroscopic failure mode of granite specimens with varying initial damage levels transitions from tensile failure to shear failure. This is also consistent with the results of Cheng et al. [27], who found that the presence of water causes the grain fracture mode of sandstone to transition from transgranular fracture to intergranular fracture. However, this study further clarifies the microscopic failure mode of mixed-mode fracture in the specimen under the coupling effect of the initial damage level and moisture state.

These findings offer important engineering guidance for evaluating the stability of surrounding rock and preventing associated geological disasters in deep rock mass engineering in water-rich conditions. First, in terms of stability evaluation parameters, this study quantifies the amplification effect of the initial damage level on the water-damage weakening effect. When the granite specimen is in the forced saturated state, the water-damage weakening coefficient *f*_n_ of the specimen with the 0% initial damage level is only 0.20, whereas that of the specimen with the 28% initial damage level reaches 0.42. This provides specific guidance for practical engineering: in stability evaluation, engineering design should adopt a higher water-damage weakening coefficient for high-damage rock masses affected by engineering disturbance, with a recommended value of *f*_n_ = 0.42. Conversely, for undamaged rock masses, stability evaluation and engineering design should adopt a lower water-damage weakening coefficient to more accurately assess the actual dynamic bearing capacity of the rock mass during instability, with a recommended value of *f*_n_ = 0.20. Second, in terms of practical engineering and mitigation measures, this study provides a reference for engineering decision-making. The experimental data show that the dynamic peak stress of the specimen with the 28% initial damage level under forced saturation is 41.6% lower than that of the specimen with the 0% initial damage level in the dry state. This significant strength deterioration provides a theoretical basis for implementing targeted mitigation measures in practical engineering. Simultaneously, when damaged and water-rich surrounding rock areas are identified through P-wave testing and hydrogeological surveys, targeted engineering interventions must be implemented in practical engineering. On one hand, advance drilling drainage or curtain grouting should be implemented in practical engineering to alter the saturation state of the surrounding rock or isolate water sources. On the other hand, in practical engineering, it is necessary to enhance the support level by means such as increasing bolt density and shotcrete strength, to compensate for the significant loss of surrounding rock bearing capacity caused by the water-damage coupling effect.

In summary, the novelty of this study lies primarily in the following: through dynamic mechanical tests on granite specimens with varying initial damage levels and moisture states, it reveals the coupled interaction mechanism of the initial damage level and moisture state on the dynamic response of rock. Unlike previous studies, this study introduces a water-damage weakening coefficient and confirms that the initial damage level exerts a significant amplifying effect on the water weakening effect. Furthermore, this study also elucidates the nonlinear energy dissipation patterns of rocks under this coupling effect and the transformation mechanism of microscopic failure modes. These research findings provide a reference for evaluating the stability of surrounding rock and preventing associated geological disasters in deep rock mass engineering in water-rich conditions.

## 5 Conclusions

In this study, we take granite from a mine in Eastern China as the research object. We conducted the SHPB test, SEM test, XRD test, and elastic longitudinal wave velocity test on granite specimens with varying initial damage levels and moisture content. We analyze the dynamic mechanical properties, water-damage weakening coefficient *f*_n_, macroscopic failure patterns, fractal dimension, energy dissipation density, and microscopic failure mode. The following main conclusions were drawn:

(1) The initial damage level and moisture content exert a significant deteriorating effect on the dynamic mechanical properties of granite. As the initial damage level and moisture content increase, the dynamic peak stress and peak modulus of granite specimens decrease. Under the 28% initial damage level and forced saturated state, the dynamic peak stress and peak modulus were reduced by 41.6% and 62.0%, respectively, compared with the dry state at the 0% initial damage level.
(2) The initial damage level of the granite specimen has an amplification effect on the weakening effect of water. The water-damage weakening coefficient *f*_n_ introduced in this study indicates that the specimen’s water-damage weakening coefficient increases as the initial damage level increases. This indicates that the initial damage level makes rocks more sensitive to water. In other words, the water weakening effect becomes more pronounced as the initial damage level increases.
(3) The water-damage coupling effect significantly alters the fragmentation characteristics and energy dissipation patterns of rocks. The quantitative analysis of the crushing morphology shows that the fractal dimension of the specimen increases from 1.801 to 1.865 as the initial damage level and moisture content increase. The energy analysis shows that at a constant initial damage level, the energy dissipation density of the specimen first increases and then decreases as the moisture content increases.
(4) The deterioration of macroscopic mechanical properties is manifested as a change in microscopic failure mode. As the initial damage level and moisture content increase, the microscopic failure mode of granite specimens transitions from transgranular fracture observed at the 0% initial damage level in the dry state, to mixed-mode fracture gradually, and finally evolves into intergranular fracture under the 28% initial damage level in the forced saturated state.

## Supporting information

S1 File Raw data supporting the dynamic peak stress variation figure. (XLSX)

S2 File Raw data supporting the Change in water-damage weakening coefficient (*f*_n_) figure. (XLSX)

S3 File Raw data supporting the dynamic peak modulus variation figure. (XLSX)

S4 File Raw data supporting the Energy dissipation density of specimens under varying initial damage levels and moisture states (A) Dry state figure. (XLSX)

S5 File Raw data supporting the Energy dissipation density of specimens under varying initial damage levels and moisture states (B) Natural moisture state figure. (XLSX)

S6 File Raw data supporting the Energy dissipation density of specimens under varying initial damage levels and moisture states (C) Natural saturated state figure. (XLSX)

S7 File Raw data supporting the Energy dissipation density of specimens under varying initial damage levels and moisture states (D) Forced saturated state figure. (XLSX)
